# Intra-articular corticosteroid injections for the treatment of people with foot and ankle osteoarthritis: a systematic review

**DOI:** 10.1093/rap/rkaf030

**Published:** 2025-03-11

**Authors:** Katherine Jones, Julie Bruce, Thomas L Lewis, Ciaran N Nolan, Shannon E Munteanu, Hylton B Menz, Michael R Backhouse

**Affiliations:** Warwick Clinical Trials Unit, Warwick Medical School, University of Warwick, Warwick, UK; Warwick Clinical Trials Unit, Warwick Medical School, University of Warwick, Warwick, UK; University Hospitals Coventry and Warwick, NHS Trust, Coventry, UK; Warwick Clinical Trials Unit, Warwick Medical School, University of Warwick, Warwick, UK; Department of Orthopaedics, Kings College Hospital NHS Foundation Trust, London, UK; Trauma and Orthopaedics, Queen Elizabeth Hospital Birmingham NHS Trust, Birmingham, UK; Department of Physiotherapy, Podiatry, Prosthetics and Orthotics, School of Allied Health, Human Services and Sport, La Trobe University, Melbourne, Australia; Department of Physiotherapy, Podiatry, Prosthetics and Orthotics, School of Allied Health, Human Services and Sport, La Trobe University, Melbourne, Australia; Warwick Clinical Trials Unit, Warwick Medical School, University of Warwick, Warwick, UK; University Hospitals Coventry and Warwick, NHS Trust, Coventry, UK

**Keywords:** foot, ankle, steroids, osteoarthritis, systematic review, corticosteroid

## Abstract

**Objective:**

Intra-articular corticosteroid injections are commonly used in the management of foot and ankle OA. Although current clinical guidelines advocate the judicious use of corticosteroid injection as an adjunct therapy, none of these recommendations are specific to the foot and ankle. Therefore, the aim of this review is to examine the effectiveness of intra-articular corticosteroid injections in people with foot or ankle OA.

**Methods:**

Four databases (Cumulative Index to Nursing and Allied Health Literature [CINAHL], MEDLINE, EMBASE and CENTRAL) and one clinical trial register (International Clinical Trials Registry Platform [ICTRP]) were searched from inception to June 2024 for randomized control trials (RCTs) and quasi-RCTs evaluating corticosteroid injection in the treatment of foot or ankle OA on pre-specified outcomes: pain, function, quality of life, safety (adverse events) and/or cost-effectiveness. Two independent reviewers conducted record screening, data extraction (Cochrane data extraction tool) and assessment of methodological quality (Cochrane Risk of Bias tool [RoB 2.0]).

**Results:**

From 1711 citations, two RCTs (57 participants, 49% males) were identified. There were no differences in pain or function over 8 weeks after a single injection of intra-articular corticosteroid compared with prolotherapy for treatment of first metatarsal joint OA. Pain and function significantly improved in people having three corticosteroid injections combined with hyaluronic acid compared with corticosteroid injections alone for treatment of post-traumatic subtalar OA. Methodological quality was graded as some concerns in both trials.

**Conclusion:**

There is insufficient evidence to guide the use of intra-articular corticosteroid injections for OA of the foot or ankle. Future robust research is needed to provide reliable evidence for this commonly performed treatment.

Key messages

- No differences after intra-articular corticosteroid compared to prolotherapy for treatment of first metatarsal joint OA.
- Pain and function significantly improved after corticosteroid injections with hyaluronic acid compared to corticosteroid alone.
- Insufficient evidence to guide the use of intra-articular corticosteroid injections for foot or ankle OA.

## Introduction

OA is a common cause of foot pain affecting one in six adults over the age of 50 [1]. Despite a recognized impact on health-related quality of life [2], this chronic, painful condition remains under researched [1–4]. A UK cross-sectional survey of community-dwelling adults aged 50 or over found that of those reporting disabling foot pain, the first metatarsophalangeal joint (MTPJ) was the most commonly affected by OA (93/525; 18%) [1]. Other joints identified in this community cohort included OA affecting the second cuneiform-metatarsal (13%), talonavicular (10%), navicular-first cuneiform (7%) and first cuneiform-metatarsal joints (4%) [1]. Multiple joint involvement is common, and three distinct radiographic phenotypes have been identified: no or minimal foot OA, isolated first MTPJ OA and polyarticular foot OA [5]. Within the midfoot, isolated medial midfoot (talonavicular, navicular-first cuneiform or first cuneometatarsal joints) OA is more common than isolated central midfoot (second cuneometatarsal joint) OA or combined medial and central midfoot OA [6]. A systematic review found that symptomatic radiographic ankle OA was less common, with prevalence of 3% of adults ≥50 years (50–64 years, 4%; 65–74 years 3% and >75 years 3%) and more common in females ≥50 years than males (4% *vs* 3%) and in those with routine/manual occupations [7].

Although OA has traditionally been characterized by osteophytes and joint space narrowing which are visible on X-ray, newer imaging techniques have identified pathological, inflammatory features such as bone marrow or cartilage lesions, subchondral cysts, joint effusions/synovitis, ligament pathology and tenosynovitis [8, 9]. Pain has a strong association with these inflammatory lesions but a weaker association with cartilage damage [10]. Inflammation also plays a pivotal role in the development of OA, contributing to nociceptive pain, and pain centralization in people with OA [10]. As such, intra-articular corticosteroids have long been used in the management of OA, primarily because of data from studies investigating knee OA, which has then been extrapolated to other joints [11]. However, some authors have cautioned against potential negative consequences of intra-articular corticosteroid use, such as risk of chondrotoxicity, infection, skin hypopigmentation atrophy, fat pad atrophy and tendon rupture [12, 13].

Despite corticosteroids being a common treatment, their use in the management of foot and ankle OA is primarily driven by clinical guidelines including evidence from treatment of other joints [14, 15]. This is increasingly regarded as problematic because the aetiology and treatment response vary between joints. A systematic review of international treatment guidelines for OA found that none included recommendations for the foot and ankle and that there was considerable variation around the endorsement of intra-articular corticosteroid injections [16]. In the UK, National Institute for Health and Care Excellence (NICE) guidance does not provide joint-specific advice but does include the foot and ankle and emphasizes the key role of non-surgical therapies in the management of OA [15]. Within this, NICE recommends that intra-articular corticosteroid injections are considered as an adjunct to support therapeutic exercise or when other pharmacological treatments have failed but acknowledge the lack of evidence for corticosteroid injections in joints other than the knee. The Osteoarthritis Research Society International (OARSI) 2019 guideline provides specific guidance for knee, hip and polyarticular OA but conclude there is insufficient evidence for other joints [14]. The OARSI guideline recommends intra-articular corticosteroids for knee but not hip OA, although a subsequent multicentre UK randomized control trial (RCT) [17] demonstrated the clinical and cost-effectiveness of intra-articular corticosteroids for hip OA, suggesting this guidance may need updating [14].

Intra-articular injection of corticosteroid is commonly used to treat foot and ankle OA in UK clinical practice [18], but current practice guidelines do not provide information specific to the foot and ankle. Our recent UK survey of healthcare practitioners who inject the first MTPJ found that, even within a single joint, clinical practice varied considerably [17]. Notably, much of this variation was between professions and professional groups, which perhaps suggests that variation is not driven by evidence but instead is a result of cultural and environmental factors as well as legal frameworks shaping practice [17].

Four previous systematic reviews investigating non-operative treatments for OA of the first MTPJ have been published [19–22]. Firstly, Zammit *et al*.’s [21] Cochrane Review from 2010 identified no trials testing injectable products for first MTPJ for the treatment of OA. This has recently been updated, and the 2024 Cochrane Review [22], which identified one trial investigating injectable products, however, found no evidence for use of corticosteroids. King *et al*.’s [19] review of non-operative management of hallux rigidus in 2016 identified six studies investigating injectable products, however only one of which tested corticosteroids. This study was a retrospective record review of patients (*n* = 365) having corticosteroid injection for different foot and ankle conditions at one English centre [23]. Only 22 participants with first MTPJ pain were injected with steroid (DepoMedrone 40 mg with 0.5% Marcaine), with ‘significant benefit’ reported at 3 months in 20 participants, although half required surgery within 2 years for ongoing pain and reduced function. The authors concluded there was limited evidence for pain relief from corticosteroid injections. Reilly *et al*. [20] searched for experimental studies of injectable corticosteroids for OA of the first MTPJ in 2020 identifying one RCT [24] and one systematic review [19]. The trial compared a single intra-articular corticosteroid injection (triamcinolone acetonide 1.0 ml) with hyaluronic acid (1.0 ml) in 37 people with early-stage painful OA of the first MTPJ on outcomes of walking and pain. There was no difference in pain or function over 3 months, although both groups improved. However, this sample also included participants with hallux valgus, and outcome data were not presented differentially by condition [25].

None of these previous reviews focused on the whole foot and ankle and are now outdated. An up-to-date, overarching review of the effectiveness of intra-articular corticosteroid injections would help inform clinical decision making for clinicians treating people with foot and ankle OA. We aimed to investigate the clinical effectiveness of intra-articular corticosteroid injections in people with foot and ankle OA.

## Methods

This systematic review was prospectively registered (https://www.crd.york.ac.uk/prospero/; CRD42023370922) and is reported in accordance with the Cochrane Handbook for Systematic Reviews of Interventions [26] and the Preferred Reporting Items for Systematic Reviews and Meta-Analyses (PRISMA) [27].

## Eligibility criteria

We included RCTs and quasi-RCTs (where the method of allocating participants to treatment was not strictly random), evaluating corticosteroid injection versus other treatment of OA in the foot or ankle joints. Conference abstracts were included if published within the past 2 years if adequate detail is provided; all other study designs were excluded. Studies were eligible for inclusion if they compared corticosteroid injection to placebo/sham, no treatment or another active treatment, including physiotherapy, rehabilitation or injection. We included studies with adult, human participants, aged over 16 years, with a clinical diagnosis of foot or ankle OA. Participants with hallux valgus were excluded from this review, due to it being a separate pathology. There were no restrictions on the clinical setting, drugs or dosage of injectate. Studies were restricted to those published in English language. Pre-specified outcomes included pain, function, quality of life, safety (adverse events) and/or cost-effectiveness. Unpublished trials and conference abstracts were included where adequate detail was provided. All timepoints were considered.

## Search strategy

Electronic databases from inception to June 2024 were searched: Cumulative Index to Nursing and Allied Health Literature (CINAHL), MEDLINE, EMBASE and the Cochrane Central Register of Controlled Trials (CENTRAL). The search strategy (Supplementary Data S1, available at *Rheumatology Advances in Practice* online) was developed by the authors, informed by search strategies used in a Cochrane Review of non-surgical treatments for OA of the first MTP joint [22] in collaboration with an information specialist (SJ) at the University of Warwick. Search strategies included combinations of MeSH terms, truncation and Boolean operators. The World Health Organization (WHO) International Clinical Trials Registry Platform (ICTRP) was searched for completed and ongoing trials (to date 06/2024). All searches were carried out by the same author (K.J.), and search results generated were exported to Rayyan, where duplicates were removed. Two independent review authors (T.L.L./C.N.N.) screened the abstracts, titles and full texts for eligible trials, recording reasons for exclusion. Discrepancies at all stages were discussed between the two review authors and resolved by consensus, or by referring to other authors (M.R.B./J.B.). None of the review authors were blinded to the study authors, institution or publication source.

## Data extraction

The Cochrane data extraction form was used independently by two review authors (K.J./M.R.B.) to extract and record information. Data extracted included: (a) general information such as title, authors and publication type; (b) methodology—study aim, trial design, inclusion/exclusion criteria, sample size, randomization method, unit of allocation, number randomized, duration of participation, losses (reasons for losses) and funding; (c) population/participant details—clinical setting, method of recruitment, age, gender, years since diagnosis, radiographic severity and other sociodemographic information; (d) treatment and comparison group(s) characteristics including number per treatment group, dosage, frequency, injection interval (e) outcome measures, including data collection timepoints, outcome definitions, assessor details, units of measurement, scales, and (f) data and analysis, missing data, imputation of missing data, unit of analysis and results. Where methodological issues were unclear or outcomes were measured but not reported, authors were contacted, and data requested.

## Data synthesis and analysis

Due to the lack of studies identified, data aggregation was not possible. Therefore, results are summarized narratively.

## Risk of bias

Two review authors (K.J./J.B.) independently assessed the methodological quality of included studies using the Cochrane ‘Risk of Bias’ tool (RoB 2.0) for RCTs, against the domains of randomization process, deviations from intended intervention(s), missing outcome data, measurement of outcome and reported results [28]. Each domain was judged as ‘low’, ‘some concerns’ or ‘high’ risk. Discrepancies were discussed between the two review authors and resolved by consensus, or by referring to a third review author (M.R.B.). We derived the overall RoB judgement from the highest classified domain, i.e. if one domain was classified as high risk this was deemed high risk overall.

## Results

### Search results

**Figure 1.**
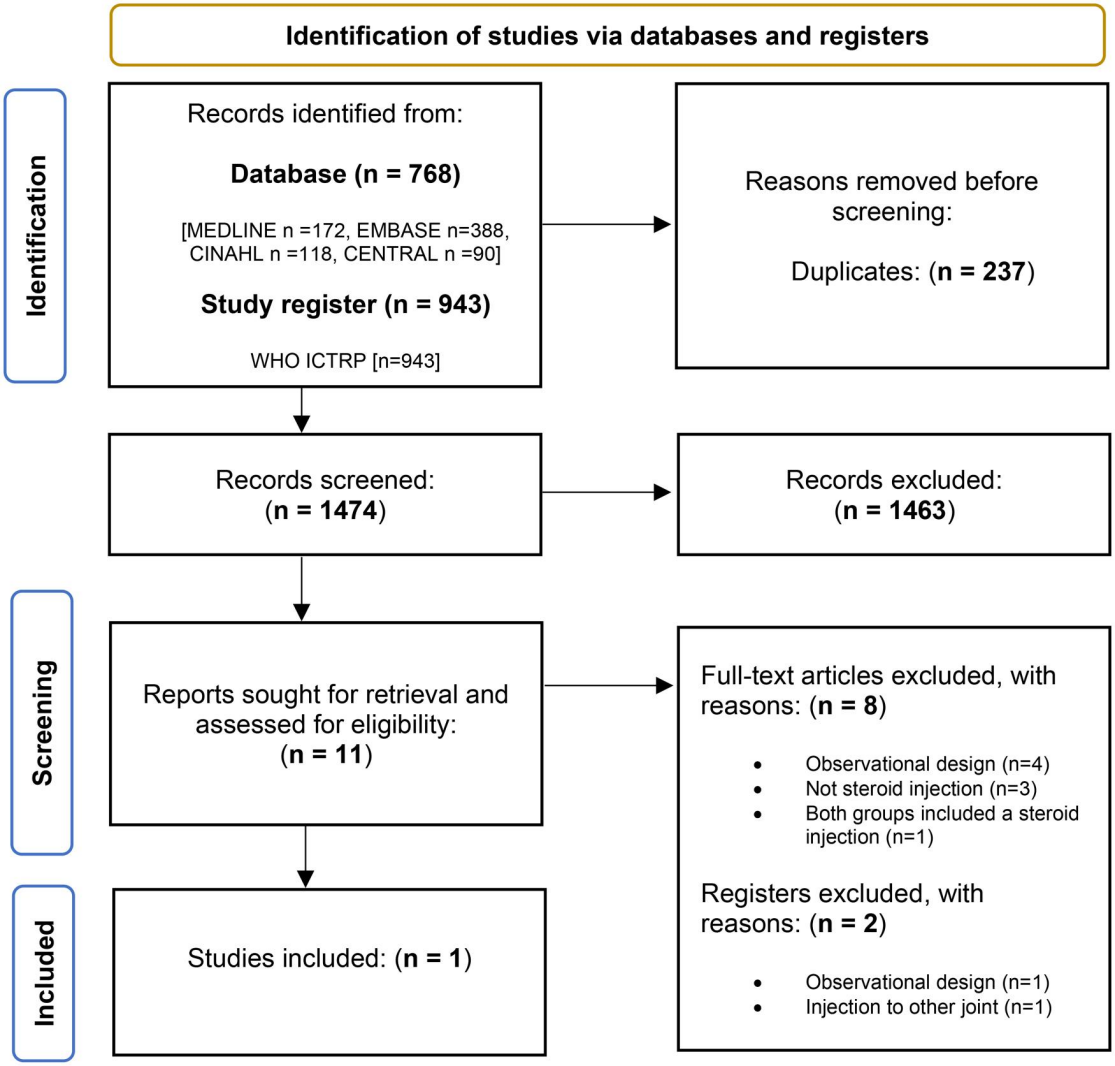
PRISMA flow diagram of literature search and study selection phases. N: number; CENTRAL: Cochrane Central Register of Controlled Trials; WHO ICTRP: World Health Organization International Clinical Trials Registry Platform

Figure 1 shows the flow diagram of the study selection process. The search strategy yielded a total of 1795 records, reduced to 1532 after excluding duplicates. After screening titles and abstracts, a further 1517 records were excluded, leaving 15 full text articles to be assessed for eligibility. Following full text screening, 13 studies were excluded for the following reasons: injections to joints other than the foot or ankle (*n* = 1), treatment other than steroid injection (*n* = 3), wrong population (*n* = 1), ongoing (*n* = 2), duplicate (*n* = 1) and observational study designs (*n* = 5) (Supplementary Data S2, available at *Rheumatology Advances in Practice* online). After full-text screening, only two trials were selected for inclusion.

### Characteristics of included studies

Characteristics of included studies are summarized in Table 1. Two small RCTs (comprising of 57 participants, 49% males), undertaken in Iran (*n* = 35) [29] and the other Brazil (*n* = 25) [30], both published in 2023, were included. The type, frequency and dosage of injections delivered varied; Hadianfard *et al*.’s [29] study ‘equally assigned’ participants to either a single corticosteroid (methylprednisolone 40 mg with 2% lidocaine) or prolotherapy (50% dextrose with 2% lidocaine) injection. Gomes *et al*. [30] randomized participants to either a single injection per week for 3 weeks of corticosteroid only (2 ml solution of betamethasone dipropionate 5 mg/ml [10 mg] and betamethasone sodium phosphate 2 mg/ml [4 mg]) or corticosteroid combined with hyaluronic acid (2 ml at 10 mg/ml).

**Table 1.** Characteristics of included studies

Author/year	Study design	Participants	Treatments	Outcome-measures/timepoint
Hadianfard *et al*. [29] Country: Iran	RCT Parallel block randomization (‘equal allocation’) Participant and statistician blinded	*N* = 32 F/M = 27/5 Mean age 48 years (range 30–65) Population: grade I or II MTP joint OA	***Active*** (*n* = 16): mixture of 1 cc methylprednisolone acetate (40 mg) and 1 cc of lidocaine 2%. Single injection	Pain (VAS 0–10cm) Function (MOxFQ 16-item) Adverse events Timepoints: baseline, 1, 4 and 8 weeks after injection; verbal completion with researcher
			***Control*** (*n* = 16): mixture of 1 cc dextrose 50% and 1 cc of lidocaine 2%. Single injection	
Gomes *et al*. [30] Country: Brazil	RCT Participant blinded	*N* = 25 F/M = 2/23 Mean age 50 years (SD 8) Population: post-traumatic subtalar OA	***Corticosteroid*** (*n* = 12): mixture 2 ml betamethasone dipropionate 5 mg/ml (10 mg) + betamethasone sodium phosphate 2 mg/ml (4 mg) total injected volume 2 ml Single injection repeated weekly for 3 weeks	Pain (VAS 0–10cm) Function (AOFAS 0–100 scale) Adverse events Timepoints: baseline, 4, 12 24 weeks after third injection; independent assessor
			***Corticosteroid + hyaluronic acid*** (*n* = 13) As per corticosteroid plus 2 ml hyaluronic acid at 10 mg/ml, total injected volume 4 ml Single injection repeated weekly for 3 weeks	

RCT: randomized controlled trial; MTPJ OA: osteoarthritis in the first metatarsophalangeal joint; MOFQ: Manchester-Oxford Foot Questionnaire; CC syringe (23 gauge); AOFAS: American Orthopaedic Ankle-Hindfoot Scale; VAS: visual analogue scale.

## Findings by outcome

### Pain

Both included trials assessed pain on a visual analogue scale (VAS) (0–10cm). The single corticosteroid or prolotherapy injection trial [29] involving participants with grade I or II OA in the first MTPJ, determined using the Coughlin and Shurnas classification, found no differences in pain scores over 1, 4 or 8 weeks (Table 2). The trial by Gomes *et al*. [30] compared corticosteroid only to corticosteroid with hyaluronic acid in participants with post-traumatic subtalar OA. This study reported significantly lower pain scores in those having corticosteroid combined with hyaluronic acid compared with corticosteroid alone at 12 (VAS 2.8 *vs* 5.8; *P* = 0.003) and 24 weeks (VAS 2.6 *vs* 6.3; *P* = 0.003), but not at 4 weeks after the third injection. Neither trial described whether pain intensity scores applied to pain at rest or when walking.

**Table 2.** Findings from included studies

Hadianfard *et al*. [29]	VAS (0–10)^a^				MOxFQ (0–100)^b^			
Timepoint	Corticosteroid Mean (SD)^c^	Prolotherapy Mean (SD)^c^	MD^d^	*P* value	Corticosteroid Mean (SD)^c^	Prolotherapy Mean (SD)^c^	MD^d^	*P* value
Baseline	6.06 (1.34)	5.68 (1.44)	0.38	0.491	49.62 (9.06)	45.37 (7.94)	4.25	0.254
1 week	2.25 (1.41)	2.50 (1.41)	−0.25	0.323	35.37 (8.17)	35.62 (7.78)	−0.25	0.930
4 weeks	2.37 (1.58)	2.68 (1.40)	−0.31	0.305	33.06 (8.29)	33.12 (7.30)	−0.06	1.00
8 weeks	2.68 (1.70)	2.81 (1.37)	−0.13	0.699	33.75 (8.49)	33.12 (7.30)	0.63	0.825
Gomes *et al*. [30]	VAS (0–10)^a^				AOFAS (0–100)^e^			
Timepoint	Corticosteroid Mean (SD)	Corticosteroid + HA Mean (SD)	MD^d^	*P* value	Corticosteroid Mean (SD)	Corticosteroid + HA Mean (SD)	MD^d^	*P* value
Baseline	6.6 (1.5)	6.9 (1.9)	−0.3	0.46	77.2 (12.2)	69.5 (19.4)	7.7	0.31
4 weeks	5.4 (1.8)	3.7 (1.7)	1.7	0.6	86.2 (14.1)	94.1 (10.9)	−7.9	0.04
12 weeks	5.8 (1.9)	2.8 (1.9)	3.0	0.003	76.7 (17.8)	93.3 (12.2)	−16.6	0.01
24 weeks	6.3 (2.1)	2.6 (2.6)	3.7	0.003	76.2 (17.6)	90.6 (19.2)	−14.4	0.02

a VAS metric 0–10, where 10 = worse pain possible.

b MOxFQ 0–100 metric, where 100 = most severe.

c Data not published.

d Unadjusted mean difference (MD) calculated by review team.

e AOFAS 0–100 metric, where 100 = healthy.

VAS: visual analogue scale; MOxFQ: Manchester-Oxford foot questionnaire; AOFAS: American Orthopaedic Ankle-Hindfoot Scale.

### Function

The effects of a corticosteroid injection on function was assessed in both trials, measured over time using the Manchester-Oxford Foot Questionnaire (MoXFQ) [29] and the American Orthopaedic Ankle-Hindfoot Scale (AOFAS) [30]. After 8 weeks, Hadianfard *et al*.’s [29] trial found no difference in function after a single corticosteroid or prolotherapy injection. Gomes *et al*. [30] reported better function after three injections of corticosteroid with hyaluronic acid compared with corticosteroid alone at all follow-up timepoints (Table 2).

### Adverse events

Both trials reported on adverse events. No complications related to the series of injections or important adverse events occurred during the study period in Gomes *et al*.’s [30] study. Hadianfard *et al*. [29] reported that no bruising, infection or complications were observed, other than a report of slight pain at the injection site, although no further data were provided.

### Additional pre-specified outcomes

Quality of life and cost-effectiveness were not reported in either of the included trials.

### Ongoing trials

We searched WHO ICTRP, which covers 18 national trial registries, for registered ongoing trials and unpublished studies. Three studies of interest were identified (KCT0008690, IRCT20210308050637N1, RBR-8t6qj75) (Supplementary Data S3, available at *Rheumatology Advances in Practice* online), one of which was already included within the review (IRCT20210308050637N1) [29] and the second confirmed by authors as complete but not yet published (RBR-8t6qj75). The last remaining study of interest (KCT0008690) was classified as completed, but attempts to obtain an update on the study progress were unsuccessful.

### Risk of bias

**Figure 2.**
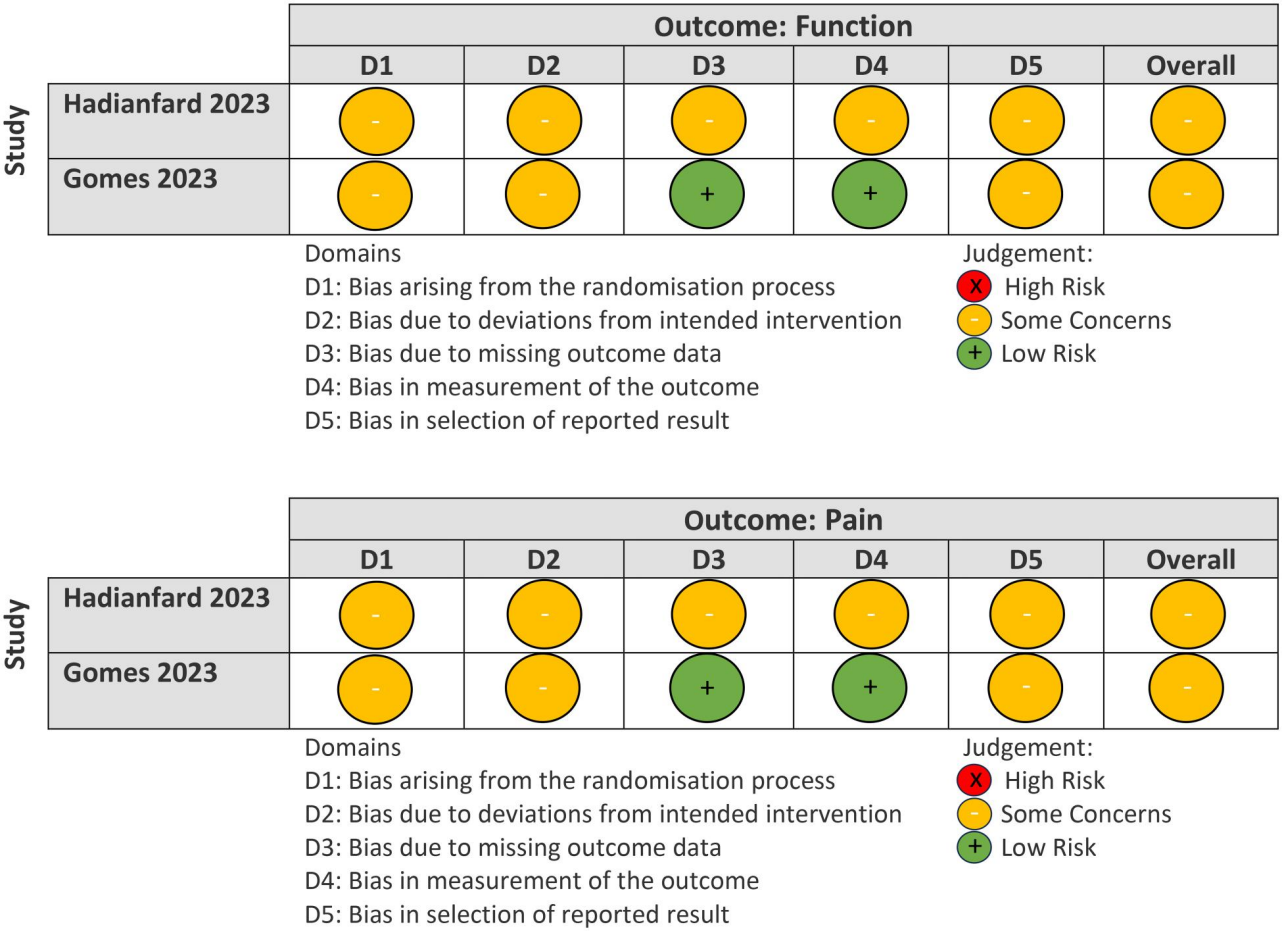
Risk of bias graph

Overall, methodological quality of the included trials was graded as some concerns (Fig. 2). Concerns arose from the randomization process in both trials because either it was not undertaken independently from investigators, or the allocation sequence was not clearly explained. Although both trials were participant-blinded, one trial [29] collected outcome data verbally by a researcher with risk of knowledge of group allocation. Concerns were also raised around missing outcome data, with one paper [29] omitting outcome data values (means, s.d.) in the main publication.

## Discussion

Intra-articular corticosteroid injections are commonly used to treat OA, both in the foot and ankle and elsewhere in the body, but clinical practice is highly variable [11, 18]. This review highlights the lack of evidence to inform clinical decision making, and even to determine the effectiveness of intra-articular corticosteroid injections in the treatment of foot and ankle OA.

We prospectively registered this systematic review and adhered to Cochrane Collaboration methodology, focusing on prespecified outcomes and eligibility criteria consistent with recommendations [26]. Our sensitive systematic search identified 1795 potentially eligible studies, but after detailed screening, only two small clinical trials met our inclusion criteria. In line with this robust methodology, we restricted the systematic review to the highest quality evidence, focusing on experimental rather than observational designs. Randomization balances known and unknown confounding factors across treatment groups, thereby enabling investigation of the causal relationship between experimental treatment, relative to comparator, and outcome [26, 31]. This reduces bias and enables the most accurate estimate of a therapy’s true effect.

The two small, single centre trials must be considered in context. Firstly, the study by Hadianfard *et al*. [29] compared methylprednisolone acetate and lidocaine with dextrose and lidocaine, and authors considered dextrose as an active comparator as it is commonly used in prolotherapy [29]. There were no differences in pain or function between treatment groups over time. Without a placebo, or non-treatment control group, it is not possible to confirm efficacy of injected corticosteroids as within group changes may be considered as regression to the mean. The sample size was small, with only 16 participants per group; thus, the trial was underpowered to detect anything but an unrealistically large between-group difference. Secondly, the study by Gomes *et al*. [30] compared hyaluronic acid with and without corticosteroid; again, the sample size was too small to detect clinically important differences in pain and function over time, with only 12 to 13 participants per group. It was not possible to determine the effectiveness of intra-articular corticosteroid injection alone against placebo.

Over recent years, there has been a growing awareness of potential detrimental effects of intra-articular corticosteroid injections including chondrotoxic effects and accelerated OA progression, subchondral insufficiency fractures, osteonecrosis and increased risk of prosthetic joint infection, although the evidence is not robust [12]. Furthermore, a recent systematic review and meta-analysis suggests that although recurrent intra-articular corticosteroid injections may provide some improvements in pain, function and quality of life up to 24 months after administration, they did not outperform placebo or other injectables at 12 and 24 months [32]. Importantly, this systematic review only included trials of knee and thumb OA, so questions remain unanswered about the effectiveness of steroid injections in foot and ankle OA.

As a result of recent evidence, advice on the use of intra-articular steroids has been tempered in clinical guidelines over recent years. For example, the 2014 NICE guidelines recommended that non-steroidal injections should be an adjunct to core treatments, whereas the current 2022 guidance recommends that they should be used for short-term pain relief only, or to support exercise when other pharmacological treatments are ineffective or unsuitable [15, 33]. The 2018 European Alliance of Associations For Rheumatology [EULAR] guidelines on the management of hand OA specifically advise *against* the use of intra-articular corticosteroid injections, except in people with painful interphalangeal joint OA [34].

Future research is needed to address fundamental questions around the use of intra-articular steroids in the foot and ankle. Key uncertainties include: whether these treatments are clinically- and cost-effective, which products should be injected, whether imaging guidance improves outcomes, whether injected corticosteroids delay time to surgical treatment or whether they increase the likelihood of adverse events, such as post-injection infection.

The main limitation of this systematic review is the small number of studies investigating the effectiveness of intra-articular corticosteroid injections in people with foot and ankle OA. This limits the conclusions which can be inferred but is a reflection of the poor evidence base for this common treatment, rather than a limitation of the methodology used in the review itself. Due to our focus being on primary research and original data we did not include secondary research such as systematic reviews in our results despite these being a hierarchically high-level evidence source, future research may want to consider this. Key strengths include the broad and sensitive search strategy and use of Cochrane methodology.

## Conclusion

This study has highlighted a lack of evidence due to low-quality underpowered trials and no placebo-controlled trials to inform intra-articular corticosteroid injections for foot and ankle OA. Future robust and adequately powered research is needed to provide reliable evidence for this commonly performed treatment.

## Supplementary Material

rkaf030_Supplementary_Data

## Data Availability

All data generated or analysed during this study are included in this published article and its supplementary material.
