# Supplementary material for: Intra-articular corticosteroid injections for the treatment of people with foot and ankle osteoarthritis: a systematic review
Source: Rheumatol Adv Pract. 2025 Mar 11;9(2):rkaf030. doi: 10.1093/rap/rkaf030 (PMC11928786; doi:10.1093/rap/rkaf030)
Supplement: rkaf030_Supplementary_Data [file rkaf030_supplementary_data.docx]

**Supplementary Material Content**

Supplementary Data S1. Search Strategy

Supplementary Data S2. List of excluded studies

Supplementary Data S3: List of ongoing trials

**Supplementary Data S1. Search strategy**

**MEDLINE**

1) exp osteoarthritis/
2) osteoarthr$.mp
3) (degenerative adj2 arthritis).mp
4) or/1‐3

5) exp Toes/

6) exp foot/
7) Metatarsophalangeal Joint/
8) metatarsophalangeal joint$.mp
9) toe$.mp

10) exp ankle/

11) ankle joint$.mp.

12) exp ankle joint/

13) rearfoot.mp

14) midfoot.mp
15) Hallux Limitus/
16) Hallux Rigidus/
17) (hallux adj2 (limitus or rigidus or flexus)).mp

18) (talocrural or subtalar or talocalcaneal or talonavicular or chopart or naviculocuneiform or intercuneiform or cuneocuboid or tarsometatarsal or Lisfranc or intermetartarsal or interphalangeal) adj (joint$)

19) or/5-18

20) 4 and 19

21) exp injections/

22) injection$.mp

23) (Joint$ adj2 injection).mp

24) or/21-23

25) 20 and 24

**CINAHL**

1) (MH "Osteoarthritis+")

2) osteoarthr*

3) degenerative N2 arthritis

4) S1 OR S2 OR S3

5) (MH "Toe Joint+")

6) (MH "Toes")

7) (MH “Foot”)

8) Metatars*

9) (MH “Ankle+”)

10) ankle joint*

11) rearfoot

12) midfoot

13) (MH "Hallux Rigidus)

14) (MH "Hallux Limitus")

15) hallux rigidus

16) hallux limitus

17) hallux N2 limitus or rigidus or flexus

18) (talocrural or subtalar or talocalcaneal or talonavicular or chopart or naviculocuneiform or intercuneiform or cuneocuboid or tarsometatarsal or Lisfranc or intermetartarsal or interphalangeal) N2 (joint$)

19) or/5-18

20) 4 and 19

21) (MH "Injections+")

22) injection$

23) joint N2 injection

24) or/21-23

25) 20 and 24

**CENTRAL**

1) MeSH descriptor Osteoarthritis explode all trees

2) osteoarthr*

3) degenerative arthritis

4) #1 OR #2 OR #3

5) MeSH descriptor: [Toes] explode all trees

6) MeSH descriptor: [Foot] explode all trees

7) MeSH descriptor: [Metatarsophalangeal Joint] explode all trees
8) metatarsophalangeal joint*
9) toe*

10) MeSH descriptor: [Ankle] explode all trees

11) ankle joint*

12) rearfoot

13) midfoot

14) MeSH descriptor: [Hallux Limitus] explode all trees
15) MeSH descriptor: [Hallux Rigidus] explode all trees

16) hallux N2 limitus or rigidus or flexus

17) (talocrural or subtalar or talocalcaneal or talonavicular or chopart or naviculocuneiform or intercuneiform or cuneocuboid or tarsometatarsal or Lisfranc or intermetartarsal or interphalangeal) N2 (joint$)

18) #5 OR #6 OR #7 OR #8 OR #9 OR #10 OR #11 OR #12 OR #13 OR #14 OR #15 OR #16 OR #17

19) #4 AND #18

20) MeSH descriptor: [Injections] explode all trees

21) injection*

22) Joint$ N2 injection

23) #20 OR #21 OR #22

24) #19 AND #23

**EMBASE**

1) exp osteoarthritis/
2) osteoarthr$.mp
3) (degenerative adj2 arthritis).mp
4) or/1‐3

5) exp Toes/

6) exp foot/
7) Metatarsophalangeal Joint/
8) metatarsophalangeal joint$.mp
9) toe$.mp

10) exp ankle/

11) ankle joint$.mp.

12) rearfoot.mp

13) midfoot.mp
14) Hallux Limitus/
15) Hallux Rigidus/
16) (hallux adj2 (limitus or rigidus or flexus)).mp

17) (talocrural or subtalar or talocalcaneal or talonavicular or chopart or naviculocuneiform or intercuneiform or cuneocuboid or tarsometatarsal or Lisfranc or intermetartarsal or interphalangeal) adj (joint$)

18) or/5-17

19) 4 and 18

20) exp injections/

21) injection$.mp

22) (Joint$ adj2 injection).mp

23) or/20-22

24) 19 and 23

| **Supplementary Data S2.** Table of excluded studies at full text with reasons | | |
| --- | --- | --- |
| **Study Title** | **Author (Year)** | **Reason for exclusion** |
| Effects of ankle joint injection combined with massotherapy on function recovery and quality of life of ankle joint osteoarthrosis patients | Qi (2018) | Wrong intervention |
| Efficacy of multimodal drug injection after supramalleolar osteotomy for varus ankle osteoarthritis: a prospective randomized study | Kim (2016) | Wrong intervention |
| Guided Intra-articular Corticosteroid Injections in the Midfoot | Protheroe (2018) | Wrong study design |
| Naviculocuneiform and second and third tarsometatarsal articulations: Underappreciated normal anatomy and how it may affect fluoroscopy-guided injections | Hansford (2019) | Wrong study design |
| Intra-articular Corticosteroid Injections in the Foot and Ankle: A Prospective 1-year Follow-up Investigation | Ward (2008) | Wrong study design |
| Fluoroscopically guided diagnostic and therapeutic injections into foot articulations: report of short-term patient responses and comparison of outcomes between various injection sites | Peterson (2011) | Wrong study design |
| Sodium hyaluronate in the treatment of hallux rigidus. A single-blind, randomized study | Pons (2007) | Wrong population |
| Short-term Efficacy of Ultrasonographic Guidance for Intra-articular Corticosteroid Injection in Hallux Rigidus: A Single-Blind Randomized Controlled Trial | Razavi (2021) | Wrong intervention |
| **ID Number/Study Title: Trial Registries** | **County** | **Reason for exclusion** |
| IRCT20100827004641N19: Comparison between clinical effects of intra-articular ozone and intra-articular steroid injection in the treatment of chronic ankle osteoarthritis | Iran | Wrong intervention |
| ISRCTN32433800: How are repeat(ed) steroid injections into osteoarthritic joints used and what are the outcomes? | UK | Wrong study design |
| IRCT20210308050637N1: Comparison of the effectiveness of 50% dextrose and corticosteroid injections in the treatment of osteoarthritis of the first metatarsophalangeal joint | Iran (registration for included trial) | Included in review |
| ICTRP: KCT0008690: A prospective randomized study on the effects of clinical use of hyaluronic acid in patients with ankle osteoarthritis lesions | South Korea | Ongoing |
| ICTRP: RBR-8t6qj75: Effect of Hyaluronic Acid Injection in the subtalar joint of patients with Osteoarthritis | Brazil | Ongoing |

| **Supplementary Data S3.** Table of ongoing studies | | | |  |
| --- | --- | --- | --- | --- |
| **ICTRP Number/ CN Number** | **Status** | **Country** | **Study Description** | **Estimated Completion** |
| ICTRP: KCT0008690/ CN-02592030; A prospective randomized study on the effects of clinical use of hyaluronic acid in patients with ankle osteoarthritis lesions | Completed *^a^* | South Korea | INTERVENTION: Drug: A standard sterile skin preparation technique was performed around the ankle joint, and intra‐articular injection was performed medial to the tibialis anterior tendon in the same location used for the anteromedial portal during ankle joint arthroscopy. Since this study was undertaken to investigate the effects of HA plus corticosteroid versus corticosteroid alonec, we decided to add HA to the conventional intra‐articular corticosteroid injection regimen. For patients in the CS+HA group, 2 ml of HA (sodium HA, molecular weight, 3000 kDa; 2 mL, Hyruan Plus®; LG Life Sciences, Iksan, Korea) and 3 ml of mixture including 1 ml of corticosteroid (2.5 mg/ml, Triam®, ShinPoong Pharmaceuticals, Seoul, Korea), 1 ml of 0.5% bupivacaine (bupivacaine HCl®, Hana Pharm, Seoul, Korea), and 1 ml of normal saline were injected on the first week, followed by single injections of 2 ml of HA on the second and third weeks. In the CS group, 3 ml of mixture including 1 ml of corticosteroid, 1 ml of 0.5% bupivacaine, and 1 ml of normal saline were injected slowly. CS: Corticosteoroid (Triamcinolone) HA: Hyaluronic acid  CONDITION: Diseases of the musculoskeletal system and connective tissue  PRIMARY OUTCOME: The Ankle Osteoarthritis Scale (AOS)  SECONDARY OUTCOME: Side effects related injection Visual analog scale  INCLUSION CRITERIA: A person diagnosed with ankle arthritis Adults aged 19 years or older who can express their opinions Person who consented to this study | August 2023 |
| ICTRP: RBR-8t6qj75/ CN-02606641; Effect of Hyaluronic Acid Injection in the subtalar joint of patients with Osteoarthritis | Recruitment Completed *^a^* | Brazil | Functional evaluation of the results of the Hyaluronic Acid Injection in the subtalar joint in patients with post-traumatic Osteoarthritis who underwent Osteosynthesis of the calcaneus with a Kirschner wire. This is a randomized, controlled, double-blind clinical study with three arms and a convenience sample size. This is a continuation of the follow-up of a previous study, which demonstrated an evaluation of subtalar infiltration after 6 months. Thus, we sought to recruit all patients of this study (n=38) to integrate the present research, whose proposal was to reassess aspects of the degree and progression of osteoarthritis after about 3 years of the infiltration protocol. The randomization of the initial study, carried out by raffle, was maintained. Therefore, this study included all patients who underwent intra-articular infiltration for the treatment of symptomatic subtalar osteoarthritis secondary to calcaneal fracture at the National Institute of Traumatology and Orthopedics Jamil Haddad between 2017 and 2018. At the time of the trauma, all patients were treated with osteosynthesis of the calcaneus using a Kirschner wire, which was the standard treatment at the institution's service. As an exclusion criterion, patients with subtalar ankylosis, previous infection, radiographic parameters compatible with inadequate joint reduction, hypersensitivity to hyaluronic acid, pregnancy and breastfeeding, concomitant diseases affecting the joint, open wound or ulcer, use of anticoagulants or altered bleeding time, intra-articular injection or arthroscopy less than 6 months and patients undergoing subtalar arthrodesis. And so, 38 patients participated in the initial study, and there was success in contacting 34 participants of them, originally allocated in the respective groups: 11 in the hyaluronic acid group (HA), 11 in the corticoid alone group (C) and 12 in the hyaluronic acid group associated with corticoid (HA+C). At least one year after the osteosynthesis, the participants received three doses of injections in the subtalar, with an interval of one week, under local anesthesia and in a sterile way. The pharmacological treatment regarded: Group HA was PolireuminR 20 mg in 2 ml (TRB Pharma, Campinas, Brazil); Group C was BetatrintaR 14 mg in 2 ml (Eurofarma, São Paulo, Brazil); and Group HA + C was 2 ml of PolireuminR + 2 ml of Betatrinta following the same doses above). Participants and researchers were blinded to the groups, except for the researcher who performed the subtalar injection. After about 3 years, the evaluation of the participants was carried out blindly. | Complete but not yet published* |
| *^a^* No results available at time of publication  *Confirmed by authors  CN, Cochrane Central Register of Controlled Trials | | | | |
